# Actualities in the Morphology and Immunohistochemistry of Cutaneous and Ocular Melanoma: What Lies Ahead? A Single-Centre Study

**DOI:** 10.3390/biomedicines10102500

**Published:** 2022-10-07

**Authors:** Andreea Cătălina Tinca, Raluca Moraru, Iuliu Gabriel Cocuz, Mihaela Cornelia Șincu, Raluca Niculescu, Adrian Horațiu Sabău, Diana Maria Chiorean, Andreea Raluca Szoke, Silviu-Horia Morariu, Ovidiu Simion Cotoi

**Affiliations:** 1 Doctoral School of Medicine and Pharmacy, University of Medicine, Pharmacy, Sciences and Technology “George Emil Palade” of Targu Mures, 540142 Targu Mures, Romania; 2 Pathology Department, Mures Clinical County Hospital, 540011 Targu Mures, Romania; 3 Department of Plastic Surgery, Mureș Clinical County Hospital, 540011 Targu Mures, Romania; 4 Anatomy Department, University of Medicine, Pharmacy, Sciences and Technology “George Emil Palade” of Targu Mures, 540142 Targu Mures, Romania; 5 Dermatology Department, University of Medicine, Pharmacy, Sciences and Technology “George Emil Palade” of Targu Mures, 540142 Targu Mures, Romania; 6 Department of Dermatology and Venerology, Mures Clinical County Hospital, 540011 Targu Mures, Romania; 7 Pathophysiology Department, University of Medicine, Pharmacy, Sciences and Technology “George Emil Palade” of Targu Mures, 540142 Targu Mures, Romania

**Keywords:** melanoma, immunohistochemistry, BRAF, metastases, sentinel lymph nodes

## Abstract

Melanoma is the most aggressive melanocytic tumor whose incidence is continuously increasing worldwide. Methods: We highlight the morphological, immunohistochemistry, and particularities of various melanoma types based on the cases diagnosed in our department from 2017 to 2021. Results: We present 100 melanoma cases and one capsular nevus case. The most common type was nodular melanoma. The immunohistochemistry markers used were SRY-box transcription factor 10 (SOX10), S100 protein, human melanoma black 45 (HMB45), and melanoma antigen recognized by T cells 1 (Melan-A). Uveal melanoma and conjunctival melanoma represent particular tumors with independent prognostic factors. Uveal melanoma requires assessment of macrophages, microvascularisation, and mitoses. Sentinel lymph node metastases are essential targets that provide staging tools. Conjunctival melanoma and capsular nevi are diagnostic pitfalls. Conclusion: Melanoma can appear in various forms, and sometimes the diagnosis might be unclear. Today, immunohistochemistry remains the most important tool in confirming the diagnosis and prognosis for this type of neoplasia.

## 1. Introduction

Melanoma is a malignant tumor that develops from melanocytes, the cells responsible for producing melanin. These cells originate from the ectoderm, mainly in the neural crest, and have a complex migratory pattern during embryo development. Therefore, their tumors can form in the skin, the mucous membranes, and the eye, where melanocytic cells are also present.

One of the most aggressive skin tumors currently known is cutaneous melanoma. This type of cancer is becoming more common, affecting people at increasingly younger ages, with a prevalence that has increased dramatically in recent years. When risk factors are present, melanoma can develop at any skin level. This tumor may develop alongside the melanocytic nevus, or it may emerge de novo. The World Health Organization (WHO) reported that one in every 33,000 nevi is a suspected melanoma [1]. The histological and immunohistochemical methods currently used in pathology laboratories and the emerging methods connected to the genetic analysis of malignant tumors represent the gold standard for melanoma diagnosis. The clinical history of the tumor has been associated with various melanoma characteristics, including the Breslow index, ulceration, mitosis incidence (which is losing relevance), inflammatory cells (lymphocytic inflammatory infiltration), and lymphatic and vascular invasions. Another significant independent prognostic factor is the presence of metastases in the sentinel lymph nodes [2,3].

The WHO categorizes cutaneous melanoma according to its association with ultraviolet (UV) exposure, often defined as cumulative solar damage. Here, we identify the melanoma types related to UV exposure (such as superficial melanoma, lentigo maligna, and desmoplastic melanoma) and those not thought to be associated with UV exposure and their impact on skin analyzers (acral melanoma, Spitz melanoma, mucosal melanoma, congenital nevi melanoma, uveal melanoma, and blue nevus melanoma). Nodular melanoma, a tumor that develops regardless of the pathway melanocytes undergo to malignant transformation, is classified as a different entity and belongs in both lists [1,4].

Uveal melanoma is the most prevalent melanoma type in the eyes. The ciliary bodies, choroid, and iris are three components that make up the uveal tract. Melanocytic nevi or neurofibromas, lymphomas, and adenocarcinomas can also be found at this level. The choroid is the area most frequently impacted among them. Conjunctival melanoma represents 5% of all ocular malignant melanocytic tumors and is another melanoma location in the eye. The conjunctiva’s melanocytes are the source of this tumor proliferation [5,6,7].

Several genetic mutations are associated with developing various melanoma subtypes and their precursor lesions. The WHO has reported several genetic mutations associated with tumors caused by low UV exposure. Dysplastic melanocytic lesions (low- or high-grade) and superficial spreading melanoma are most commonly associated with the v-raf murine sarcoma viral oncogene homolog B1 (*BRAF*) p.V600E mutation or neuroblastoma-RAS (*NRAS*) mutations, and less often with mutations in tumor protein p53 (*TP53*), phosphatase and tensin homolog (*PTEN*), or cyclin-dependent kinase inhibitor 2A (*CDKN2A*). BAP1-inactivated melanocytoma is associated with *BRAF* or *NRAS* and BRCA1-associated protein 1 (*BAP1*) mutations. Melanoma arising from this type of profile is rare. For deep penetrating melanocytoma, *BRAF* or *NRAS* mutations are associated with Catenin Beta 1 (*CTNNB1*) mutations. Tumors associated with high UV exposure, such as melanoma in situ, lentigo maligna, and desmoplastic melanoma, are most commonly related to mutations in *BRAF* (other than p.V600E), neurofibromatosis type 1 (*NF1*), and Ras-related C3 botulinum toxin substrate 1 (*RAC1*) [1,4,5,7].

The most important modern tool in diagnosing melanoma is immunohistochemistry (IHC). This laboratory method permits the staining of certain cell types using antibodies. The IHC profile of these tumors shows positivity for a range of markers: S100 (nuclear marker; sensitive for melanoma), SRY-box transcription factor 10 (SOX10; nuclear marker; highly specific for melanoma), human melanoma black 45 (HMB45; cytoplasmic marker), and Melan-A (cytoplasmic marker). The proliferation index Ki-67 can provide further information about tumor activity, helping diagnose malignant neoplasia [8,9,10].

New methods have been developed in recent decades. Many opportunities arose with the discovery of *BRAF*. Most melanoma cases have the p.V600E mutation, the most common gene mutation. *BRAF* is mutated in almost half of all melanoma cases [11,12]. Currently, targeting the tumor microenvironment is topical. A protein called programmed cell death 1 (PD-1) promotes self-tolerance by restricting the activation and multiplication of T cells. It has been shown that the PD-1 ligand (PD-L1) is a homolog of the B7-1/B7-2 proteins. As an immune system regulatory mechanism, PD-L1 is present on epithelial, endothelial, and T lymphocytes and is expressed in various tumor types. The first immunotherapy medication against these proteins was licensed in 2014. The outcomes after the new medication (nivolumab) were significantly better than those after the classical therapy, regardless of whether chemotherapy or BRAF inhibitors were used [12,13,14,15].

In addition to PD-L1, one new protein targeted in melanoma is V domain Ig containing a suppressor of T-cell activation (VISTA), a PD-L1 homologue, which targets myeloid tumoral and inflammatory cells and is being studied worldwide to gain more information about its expression. However, current data are contradictory. While some studies have reported its expression in leukocytes and association with better prognoses, others have reported that it is expressed in tumoral cells, worsening patient outcomes. Nevertheless, the tumor microenvironment has been proven to be important for tumor development and also in the treatment response [16,17,18,19].

In this study, we aim to share our results regarding the types of cutaneous and ocular melanoma diagnosed in our hospital. We wish to present the morphological appearance and immunohistochemical profile of the following entities: nodular melanoma, superficial spreading melanoma, nevoid melanoma, uveal melanoma, and an additional rare case represented by conjunctival melanoma. For the part of cases in which we had the possibility for follow-up, we were able to diagnose the following rare entities: in-transit melanoma, capsular nevus (pitfall diagnosis for patient with sentinel lymph node resection), and intestinal metastasis. We are highlighting the importance of properly establishing the parameters used in assessing the outcome of the patients and we raise the problem of discussing the differential diagnosis, which is raising challenges. Our experience with melanoma can underline the particularities of each tumor analyzed and, furthermore, show diagnostic clues for each case.

## 2. Materials and Methods

We retrospectively selected 100 cases, of which 90 were primary cutaneous melanoma and 10 were ocular melanoma (9 uveal and 1 conjunctival). An additional case, represented by capsular nevus, was chosen to be described as a pitfall diagnosis. Eleven metastases were found, of which seven involved the sentinel lymph nodes, two involved the liver, one the small bowel, and one was in-transit metastasis. All cases were diagnosed in the Pathology Department of the Clinical County Hospital Mureș in Târgu Mures, Romania, from 2017 to 2021.

The inclusion criteria were: primary cutaneous melanoma stages pT1a–pT4b, primary ocular melanoma stages pT1a–pT4b, positive sentinel lymph nodes (including capsular nevus for debating a pitfall diagnosis), in-transit melanoma metastases, and melanoma metastases in patients in which follow-up and re-excision were performed at our hospital.

We excluded patients diagnosed with other tumor types, melanoma in situ, or premalignant melanocytic skin lesions.

A standard HP method was used to collect and process tissue samples for laboratory diagnostics. Before hematoxylin-eosin staining, samples were embedded in paraffin and preserved in 10% neutral buffered formalin. IHC analysis was performed on 4 m-thick sections of formalin-fixed, paraffin-embedded tissue using an automated immunostainer (Benchmark GX; Ventana Medical Systems, Inc.; Tucson, AZ, USA). The instructions on the package inserts for each antibody were used to determine the reagents and incubation times. The OmniMap 3,3′-diaminobenzidine detection kit (Ventana Medical Systems, Inc.) was used to create the slides, and hematoxylin was used as a counterstain. (project identification code: 19059/03.01.2022).

## 3. Results

### 3.1. Epidemiologic Data and Tumor Classification

We included 90 cases of primary cutaneous melanoma and 10 cases of ocular melanoma. One unique case of capsular nevus was selected as a representative for a pitfall diagnosis. Of the ten cases of ocular melanoma, nine were uveal melanoma and one was conjunctival melanoma. Patients presented ages from 25 (female) to 90 (male), with a mean of 62.6 and a standard deviation of 14,440. For cutaneous melanoma, we had 45 females and 45 males. For ocular melanoma, we had five females and five males.

The most common type of cutaneous melanoma was nodular melanoma (76 cases; 76%), followed by superficial spreading melanoma (12 cases; 12%) and nevoid melanoma (2 cases; 2%). There were 29 (29%) patients with pT4b stage tumors, 17 (17%) with pT1a stage tumors, 13 (13%) with pT3b stage tumors, 12 (12%) with pT4a stage tumors, 9 (9%) with pT2a stage tumors, 3 (3%) with pT1b stage tumors, and 3 (3%) with pT1a stage tumors.

We identified 11 metastases (11% of cases), of which 11 (63.63%) occurred in the sentinel lymph nodes. Unspecified skin (2; 18.18%), liver (2; 18.18%), intestinal (1; 9.09%), forefoot (1; 9.09%), calf (1; 9.09%), and in-transit (1; 9.09%) were the areas involved. The nodular melanoma subtype was associated with 10 of the 11 metastases identified, while the superficial subtype was associated with 1. The nodular melanoma cases were included in stages pT3b, pT4a, and pT4b. Superficial spreading melanoma had stage pT1a.

### 3.2. Histology and IHC

We analyzed the melanocytic tumors with the usual hematoxylin-eosin stain and IHC as a diagnostic and prognostic method. All melanoma cases required IHC for diagnosis confirmation (Table 1).

#### 3.2.1. Cutaneous Melanoma

- Nodular melanoma

**Figure 1:**
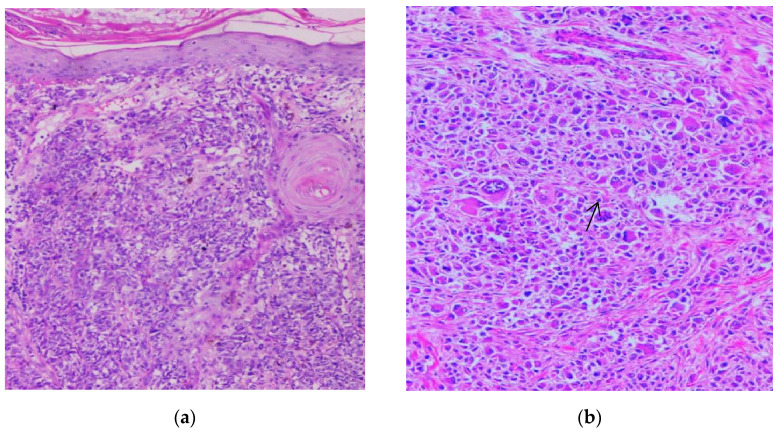
Nodular melanoma. (**a**) Nests and plaques of tumor cells can be observed at 5× magnification. (**b**) Cytological atypia (highly pleomorphic tumor cells with prominent nucleoli) can be identified. Mitoses can be observed (arrow). 20× magnification. (Collection of Pathology Department SCJ Mureș).

**Figure 2:**
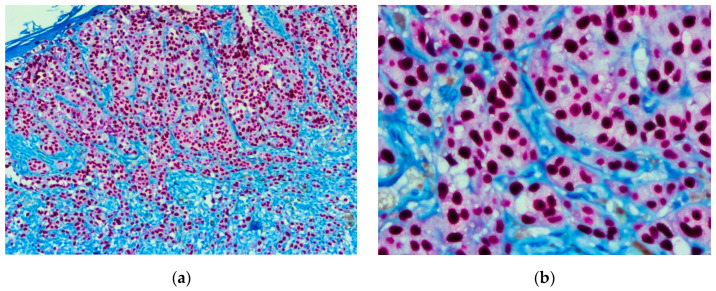
Nodular melanoma. (**a**) IHC stained with nuclear marker SOX10 (red chromogen), 5× magnification. (**b**) IHC stained with nuclear marker SOX10 (red chromogen), 40× magnification. (Collection of Pathology Department SCJ Mureș).

**Figure 3:**
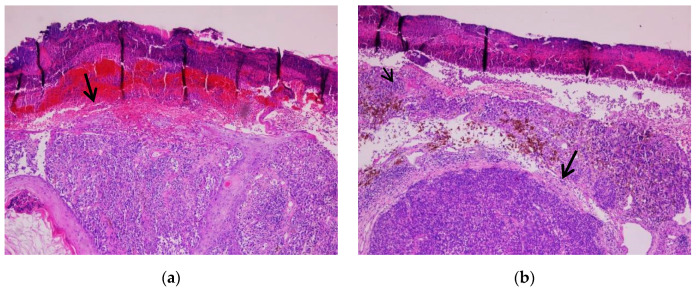
Nodular melanoma. (**a**) Ulcerated epidermis. A tumoral proliferation with solid architecture is seen underneath (arrow). (**b**) Ulcerated epidermis. Nodules of tumoral cells were observed beneath the ulceration (large arrow). Melanophages and pigmented tumoral cells (small arrow) are also evident. Both images were taken at 5× magnification. (Collection of Pathology Department SCJ Mureș).

The histological appearance of nodular melanoma in standard hematoxylin-eosin staining showed the presence of tumor proliferation with a solid architecture, extending from the surface epithelium to the dermis and occasionally the hypodermis. With a low objective (5×), we observed plaques and nests with an infiltrative pattern. Cytological features were observed at higher objectives (20×) and included medium-to-large tumoral cells with distinct cell borders and various forms (oval, round, and polygonal). The tumor cells had eosinophilic or pale cytoplasm. The nuclei were large, pleomorphic, and hyperchromic, with prominent nucleoli that had a distinctive eosinophilic color. Typical and atypical mitoses were observed in all cases. Brisk inflammatory infiltrate was present in all cases. Pagetoid migration was observed in 11 cases (14.47%). Ulceration was observed in 40 cases (52.63%). Neural and vascular invasion were observed in nine cases (11.84%), two with neural invasion and seven with vascular or lymphatic invasion. IHC showed positivity for markers SOX10, S100, HMB45, and Melan-A in all cases, with the highest intensity with nuclear marker SOX10, expressed in 90% of cells (Figure 1, Figure 2 and Figure 3).

- Superficial spreading melanoma

**Figure 4:**
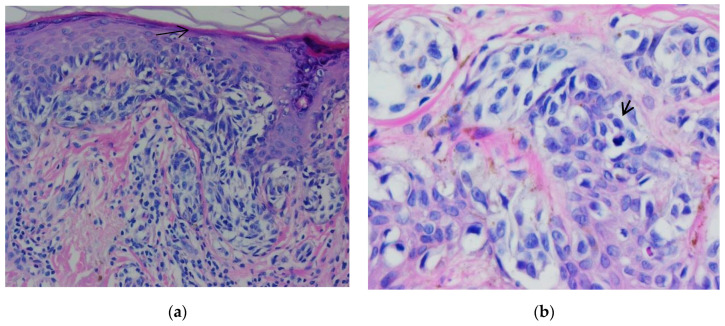
Superficial spreading melanoma. (**a**) Asymmetric tumor proliferation is observed beneath the epidermis (10× magnification). The tumoral cells are organized in nests. They are seen at the junction and extending into the depth of the dermis. Pagetoid migration is observed (arrow). (**b**) Tumor cells are organized into nests and show high atypia (40× magnification). Mitoses are seen (arrow). (Collection of Pathology Department SCJ Mureș).

We observed a solid tumor proliferation extending into the superficial dermis with superficial spreading melanoma. The tumor cells were organized into nests. They presented eosinophilic cytoplasm with pleomorphic nuclei and prominent nucleoli. Pagetoid migration was a constant feature in all 12 cases. Ulceration was observed in two cases. IHC showed positivity for SOX10, S100, Melan-A, and HMB45 markers (Figure 4).

- Nevoid melanoma

Nevoid melanoma cases presented as solid tumors with tumoral cells organized in nests and cords. One of the cases presented with a “puffy shirt” appearance of the lesion. “Puffy shirt” represents an aspect in which we can observe crowded melanocytic cells (areas of hypercellularity in which the cells seem to not have enough space). The other case presented no particularities of the architecture.

The cells in both cases appeared medium-sized, round to oval in shape, and with moderate-to-abundant cytoplasm. There were many different nuclei types; some were uniform, round, and centrally located, whereas others were pleomorphic, larger, hyperchromic, with prominent nucleoli. Mitotic activity was present in both.

**Figure 5:**
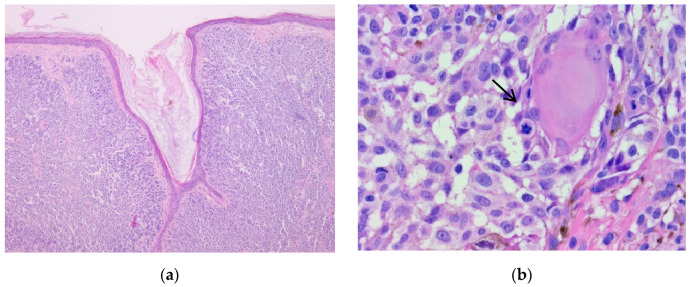
Nevoid melanoma. (**a**) Tumor architecture, 5× magnification. (**b**) Deep area of the tumor showing lack of maturation. Enlarged, atypical tumoral cells are observed, presenting prominent nucleoli. Mitoses are highlighted in the field (arrow), 40× magnification. (Collection of Pathology Department SCJ Mureș).

**Figure 6:**
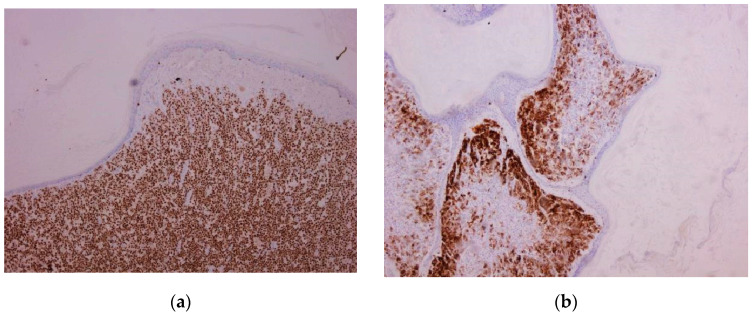
Nevoid melanoma. Tumor architecture at (**a**) 5× magnification showed by SOX10 stain and (**b**) 10× magnification IHC stain with MelanA. (Collection of Pathology Department SCJ Mureș).

**Figure 7:**
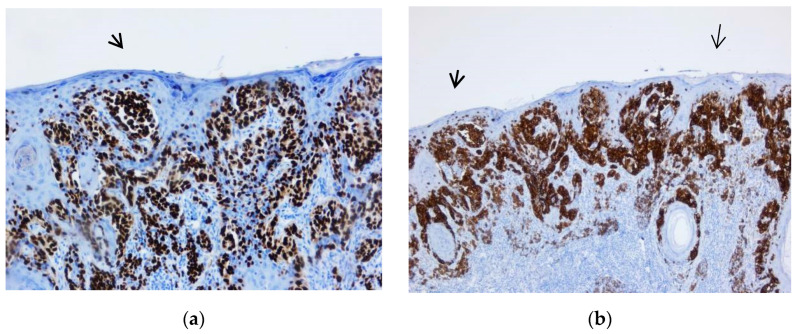
Nevoid melanoma. (**a**) IHC staining with SOX10 (10× magnification). (**b**) IHC staining with Melan-A (5× magnification). Pagetoid migration is observed, and tumor cells reach the corneous layer (arrows). (Collection of Pathology Department SCJ Mureș).

Pagetoid migration was observed in one of two cases. The maturation phenomenon was absent (one case) or minimal (one case). The tumor showed positivity for SOX10, S100, and Melan-A and intensely expressed HMB45 in the pleomorphic component (Figure 5, Figure 6 and Figure 7).

#### 3.2.2. Ocular Melanoma

- Conjunctival melanoma

A 51-year-old male patient presented with a tumor in the caruncula. It is the only case diagnosed in our hospital.

**Figure 8:**
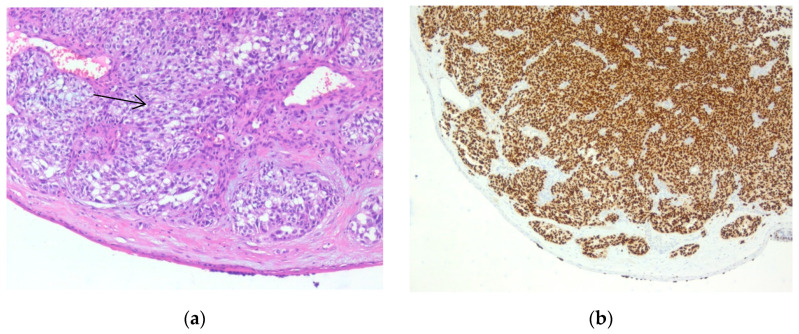
Conjunctival melanoma. (**a**) Tumor proliferation with nesting appearance extending in the conjunctiva (arrow). (**b**) IHC stain with nuclear marker SOX10. Both images were taken at 5× magnification. (Collection of Pathology Department SCJ Mureș).

On hematoxylin-eosin staining, a proliferation of tumor cells of various sizes replacing the conjunctiva’s normal epithelium was observed in the epithelial layer and below. The enlarged, hyperchromic, pleomorphic nuclei and abundant eosinophilic or pale cytoplasm gave the cells an epithelioid appearance. Mitoses were evident. The IHC markers SOX10, S100, Melan-A, and HMB45 were positive, and Ki-67 was expressed in 40% of cases (Figure 8).

- Uveal melanoma

**Figure 9:**
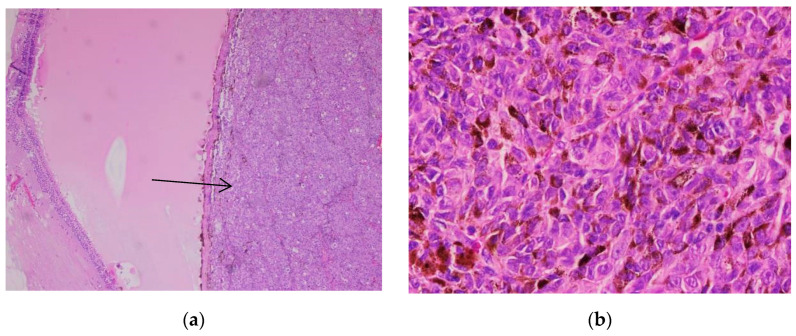
Uveal melanoma. (**a**) Solid tumor proliferation (arrow), 10× magnification. (**b**) Cytological features are seen (pleomorphic, enlarged nuclei with prominent nucleoli). Melanin pigment is observed. 40× magnification. (Collection of Pathology Department SCJ Mureș).

**Figure 10:**
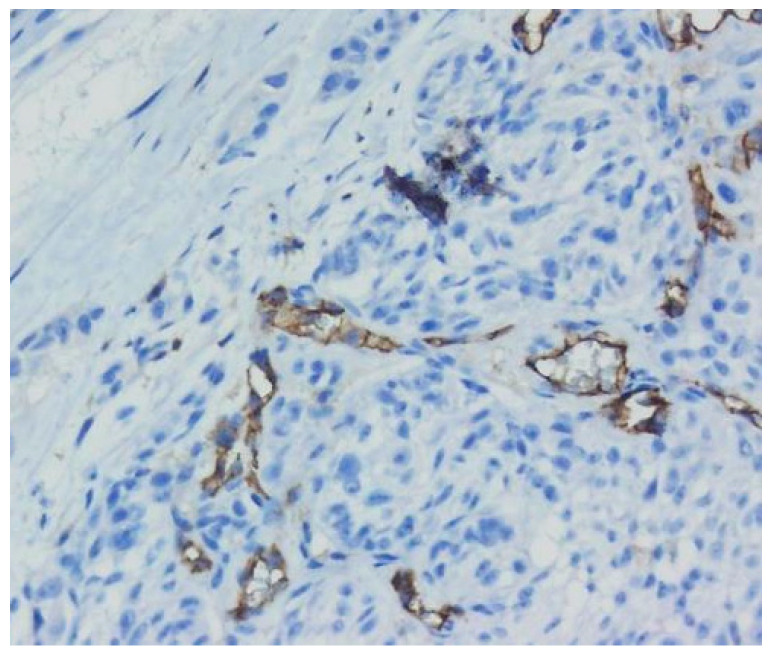
Uveal melanoma. Quantification of microvascularisation using CD31 immunostaining. 20× magnification. (Collection of Pathology Department SCJ Mureș).

Uveal melanoma cases presented as tumor proliferation comprising sheets of epitheloid and mixt tumoral cells (epitheloid and fusiform) with eosinophilic cytoplasm and pleomorphic nuclei. Stages included: pT1a (two cases), pT2a (one case), and pT3a (six cases). In all cases, macrophages were quantified using cluster of differentiation 68 (CD68) immunostaining. The tumor cells were positive for SOX10, S100, Melan-A, and HMB45. Ki-67 was expressed in all cases in percentages between 10% and 40%. The macrophages were present in moderate (seven cases) or low (two cases) quantities in the examined cases. Tumor thickness was measured in diopters (DD), varying from 6.25 to 40 DD (1 DD equals 2.5 mm). Vessel density varied from 10 to 38 mm^2^ based on immunostaining for cluster of differentiation 34 (CD34) and 31 (CD31). Mitoses counts were assessed in 40 high power fields (HPFs) and varied between 5 and 38 (Figure 9 and Figure 10).

#### 3.2.3. Sentinel Lymph Nodes and Capsular Nevi

Eleven cases presented metastases. Two were found in the liver, one in the small bowel, and one in transit. Seven involved the sentinel lymph nodes. The tumor cells had irregular shapes, eosinophilic cytoplasm, and pleomorphic nuclei. These cells were observed organized into nests and clusters or isolated and extended from the capsule towards the depth of the lymph nodes. IHC showed positivity for SOX10 (following the protocol for sentinel lymph nodes according to AJCC 8th edition).

**Figure 11:**
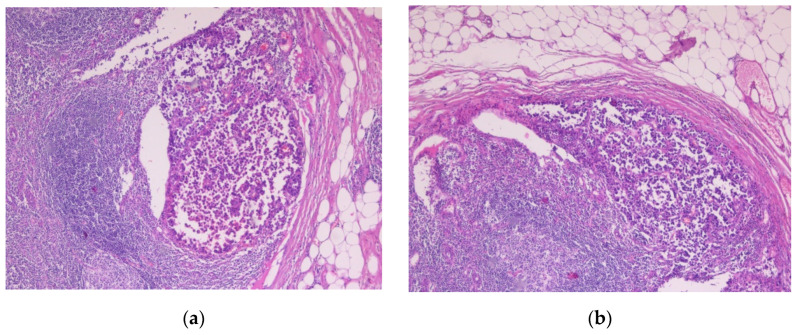
Lymph node metastasis. (**a**,**b**) Tumor proliferation below the lymph node capsule, extending towards the center of the lymph node. Both images were taken at 5× magnification. (Collection of Pathology Department SCJ Mureș).

**Figure 12:**
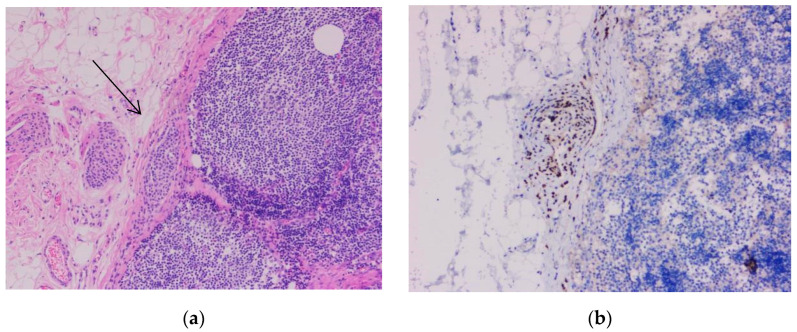
Capsular nevus. (**a**) Melanocyte proliferation in the lymph node capsule. The cells are round or ovoidal in shape, with eosinophilic cytoplasm and round nuclei located in the center of the cell (arrow). (**b**) Nuclear immunostaining with marker SOX10, showing the melanocytic cells aligned in the lymph node capsule. Both images were taken at 10× magnification. (Collection of Pathology Department SCJ Mureș).

**Figure 13:**
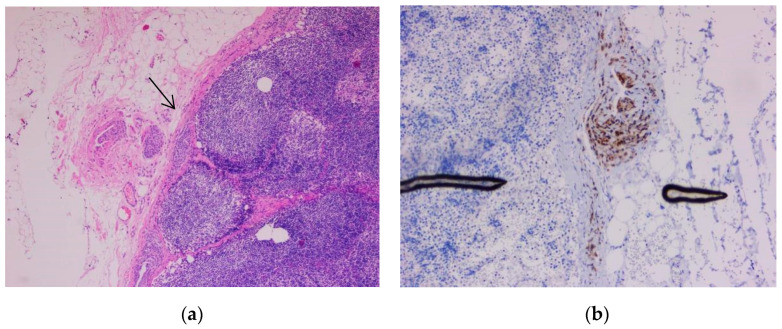
Capsular nevus. (**a**) Melanocyte proliferation in the lymph node capsule. The cells were round or ovoidal in shape, with eosinophilic cytoplasm and round nuclei located in the center of the cell, 5× magnification (arrow). (**b**) Cytoplasmic immunostaining with marker Melan-A showing the melanocytic cells aligned in the lymph node capsule (10× magnification). (Collection of Pathology Department SCJ Mureș).

One case selected in addition represented a pitfall diagnosis. A 41-year-old male patient previously diagnosed with nodular melanoma underwent sentinel lymph node excision. In hematoxylin-eosin staining, we observed a proliferation of melanocytic cells in the lymph node capsule. The proliferation measured 2.93 mm in length and 0.22 mm in width. The cells had a round shape, medium size, and eosinophilic cytoplasm with central nucleolated nuclei. Atypia was not evident. Pleomorphism was absent. Mitoses were not observed. The melanocytic cells showed positivity for immunomarkers SOX10, S100, and Melan-A but were negative for HMB45. The Ki-67-based proliferation index was expressed <1% (Figure 11, Figure 12 and Figure 13).

**Figure 14:**
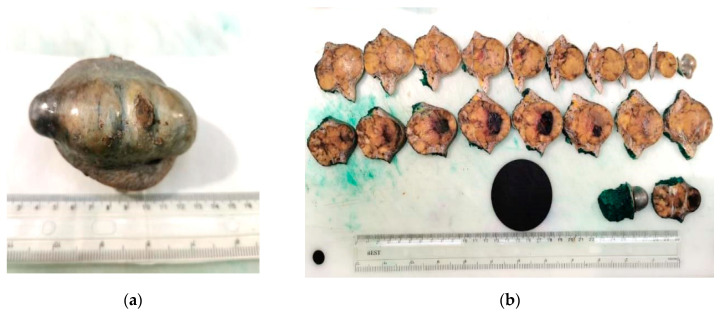
In-transit metastasis. (**a**) Gross examination. (**b**) Gross examination, cut section. We observed a well-defined nodule in the dermis and subcutaneous fat. (Collection of Pathology Department SCJ Mureș).

**Figure 15:**
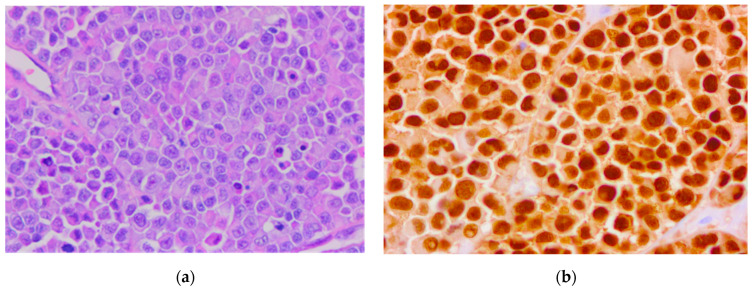
In-transit metastasis. (**a**) Hematoxylin-eosin staining. (**b**) Immunostaining with SOX10. Both images were taken at 40× magnification. (Collection of Pathology Department SCJ Mureș).

An 80-year-old male patient previously diagnosed with stage pT4b melanoma underwent surgical excision of a cutaneous tumor located distantly from the primary tumor. In hematoxylin-eosin staining, we observed a tumor proliferation of epitheloid cells with pleomorphic nuclei located in the dermis. We performed IHC for SOX10, S100, HMB45, and Melan-A to confirm the diagnosis. All markers were expressed by tumor cells. The proliferation marker Ki-67 was expressed in 80% of the malignant cells (Figure 14 and Figure 15).

We investigated the correlation between parameters using Spearman’s rank correlation coefficient (*r*_s_). Patient’s age was positively correlated with their Breslow index (*r*_s_ = 0.350; *p* = 0.0001), Clark level (*r*_s_ = 0.110; *p* = 0.0020), ulceration (*r*_s_ = 0.360; *p* = 0.0002), mitosis count (*r*_s_ = 0.170; *p* = 0.0020), and tumor stage (correlated with Breslow index). In addition, ulceration, metastases, and mitosis count were correlated with high Breslow index (*r* = 0.356; *p* = 0.0001).

## 4. Discussion

Malignant tumors such as melanoma develop more often in older males. The data cover both cutaneous and ocular melanoma. In this study, we had 50 patients (50%) of each gender, while the additional capsular nevus case was from a male individual. Five (5%) patients with ocular melanoma were male, and five (5%) were female. There were 45 (50%) male and 45 (50%) female patients diagnosed with cutaneous melanoma; therefore, a gender difference was not seen in our patients.

Previous studies have reported a mean age of onset at 57 years, with older patients presenting a greater mortality rate. The mean age of all patients in this study was 62.6 years. The information gathered for our study is consistent with the literature data. A 24-year-old female was the youngest patient, and a 90-year-old male was the oldest.

In cases of familial melanoma, the data may vary. Twenty-three families affected by melanoma were the focus of the study conducted by Goldstein [20], who examined the disease’s traits, finding males and females who experienced onset at an average age of 33 and 29 years, respectively. The tumors developed much sooner compared with patients who did not show a familial background [21,22,23].

The most common melanocytic lesion type diagnosed in our study was nodular melanoma. Studies made on large cohorts in different parts of the world, during a long period of time, have described superficial spreading melanoma as being the most common type of melanoma encountered in countries such as Germany, Italy, or the United States. In Australia, the most common type was nodular melanoma [24,25]. The most common tumor stage identified was pT4b (ulcerated nodular melanoma with a Breslow index (thickness/depth) >4 mm), followed by pT1a (melanoma with maximum thickness <1 mm and without surface epithelium ulceration). Looking at the results and observing the late stage of diagnosis for most patients, the need to identify a possible cause for this situation is mandatory. We can assume that the patient’s presentation time is mostly responsible for diagnosing melanoma in a late stage. Potential issues in the delay are the absence of clinical symptoms or the location of the tumor [24].

We found 11 metastases among the 100 patients studied. One was associated with superficial melanoma, and ten were linked to nodular melanoma. Sentinel lymph nodes were involved in most (seven) patients. All cases involving the lymph nodes were linked with nodular melanoma, staged pT3b, pT4a, and pT4b. The presence of metastases is correlated with the tumor stage, as our results also prove. The more advanced the tumor is, the higher is the probability of distant metastases [25,26].

The analysis of the sections from nodular melanoma patients showed that most of these tumors presented solid architecture with (42 patients) or without ulceration of the surface epithelium. Most tumor cells were organized in nests and presented an epithelioid appearance. The epithelioid cells were observed in 93 of the 100 patients, of which 84 had cutaneous melanoma and 9 had ocular melanoma. Mixed histological features were also reported in uveal melanoma, where both epithelioid and fusiform aspects were evident. The information in the data is consistent with previously reported descriptions and classic morphological criteria [27].

Nevoid melanoma is a specific type of melanoma, which represents a diagnostic challenge. The data on this tumor remain unclear, mainly because its diagnostic criteria are not uniformly accepted. Zhang [28] found that many of these tumors are associated with *BAP1* mutations, compared with nodular melanoma or superficial spreading melanoma, which are associated with *BRAF* and *NRAS* mutations. A diagnostic clue regarding nevoid melanoma can be the recurrence of a previously known “nevus” on the skin. The sections must be analyzed carefully, mainly because some changes can be very subtle. “Puffy shirt” is a feature in the architecture of the tumor, often seen in nevoid melanoma. In such cases, the cells look crowded, as if there is no space for all. Such areas of hypercellularity in what we think to be a benign melanocytic lesion prompts a careful evaluation of the tumor. When not observed, cytological atypia and lack of maturation (seen in benign lesions such as nevi) should raise suspicion. Once the atypia is identified, IHC is recommended in order to elucidate the diagnosis. Expression of “melanoma cocktail” (SOX10, S100, HMB45, and MelanA) along with ki67 remain vital for diagnosing nevoid melanoma.

The presence of metastases in the sentinel lymph node of a melanoma patient is an important independent prognostic factor that can change their therapy and outcome. However, the diagnosis becomes complicated when other melanocytic lesions are present in the lymph nodes. The American Joint Commission on Cancer (AJCC) recommends that sentinel lymph node excision be performed in patients who are at least at stage pT1b (a Breslow index <1 mm and ulcerated lesions) [28].

The diagnostic pitfall was represented in this study by capsular nevus. Only limited data in the literature provide information on the prognosis in such cases, yet they are stating that patients diagnosed with nevi of the sentinel lymph nodes do not require additional interventions or therapies. The five-year survival rate of patients with capsular nevi is the same as that of patients with no lesions in the sentinel lymph nodes [29].

The histology of these lesions can vary. The analysis can be difficult when the lesion is very small or composed of small nests of melanocytic cells. When we encounter such lesions, IHC becomes an invaluable tool. Although both melanoma and nevi stain positive for S100 and SOX10, markers such as Melan-A, HMB45, and Ki-67 can help differentiate them [30]. In this study, the most important diagnostic factors were the absence of visible atypia with hematoxylin-eosin staining and negative staining for HMB45 and Ki-67. HMB45 is a cytoplasmic marker that strongly stains melanoma cells, while the proliferation index is usually marked in the tumor. Another useful marker in such cases is preferentially expressed antigen in melanoma (PRAME), a newly used marker expressed in primary and metastatic melanoma but rarely in benign melanocytic lesions [31,32,33].

A study by Lezcano [34] analyzed *PRAME* expression in 30 nodal nevi, all staining negative. The same study examined the expression of the same marker in metastatic melanoma using 15 lymph node metastatic cases, finding the malignant melanocytic cells stained positive in all cases. The existence of a small number of cases diagnosed worldwide is causing a delay in the elaboration of protocols regarding these rare entities.

In-transit metastases represent melanoma nodules distant from the primary tumor site that do not involve the sentinel lymph nodes or other organs. They are believed to be caused by the spreading of tumor cells via the lymphatic system and appear in 5%–10% of melanoma patients. This type of metastases appears in advanced cases, from stage pT3a upwards, or clinical stages III and IV. Their prognosis is poor, with a 5-year survival rate of between 25–50%. The major challenge in such cases is the lack of standardized management. In this study, our specimen presented solid tumor proliferation with tumoral cells organized into nests. The cells had a round or ovoidal shape and were large, with pleomorphic nuclei and prominent eosinophilic nucleoli. The most important differential diagnoses in such cases, especially at the cutaneous level, include poorly differentiated carcinomas and sarcomas. Considering our patient’s history, we performed IHC for marker SOX10, one of the most specific markers for melanoma. Diffuse and intense staining confirmed our diagnosis [35,36,37,38,39].

A poorly investigated tumor can also be part of the differential diagnosis spectrum when encountering primary or metastatic tumors that resemble melanoma. Called melanocarcinoma, this tumor represents a combination of melanoma and squamous cell carcinoma. Only a few cases have been described to date. One was investigated by Kochoumian [40], who described a 78-year-old male patient presenting with a tumor on the forearm. The tumor consisted microscopically of two distinct cell types: pigmented epithelioid and spindle tumor cells and polygonal eosinophilic cells showing keratinization. Melanocytic markers (S100, Melan-A, and HMB45) stained the pigmented atypical cells, while anti-cytokeratin monoclonal antibodies AE1 and AE3 stained the polygonal squamoid cells.

Conjunctival melanomas are very rare tumors involving this specific part of the eye. Usually, the melanocytic lesions encountered in this area are nevi. Conjunctival melanomas presenting as lesions are most often found in the caruncula [40,41], where the tumor in our case was also observed. The presence of melanoma in this particular area is associated with a worse prognosis compared to other locations. A Finnish study by Tuomaala et al. [42] described the prognosis and incidence of conjunctival melanoma in the white population of Finland, concluding that local recurrence, tumor location, and tumor size are directly related to the mortality rate. Our patient underwent chemotherapy and refused enucleation. Follow-up, a year after diagnosis, showed no metastases or recurrence.

Uveal melanomas are unique melanocytic tumors, which can involve the iris, ciliary bodies, and the choroid. This type is associated with severity and invasion directly proportional to the tumor stage. One particularity in this neoplasm is the different perspective on the tumor microenvironment, developed in the most recent editions of worldwide guidelines (World Health Organization and American Joint Commission on Cancer) [1,28]. Unlike cutaneous melanomas, lymphocytes are quantified together with macrophages. Some studies, such as the one by Bronkhorst, highlight the changes the inflammatory cells undergo before and after therapy [43]. Macrophages show an M2-like phenotype associated with monosomy 3 (single-copy loss of chromosome 3). These cells, along with highly important macrophage attraction molecules, cause an influx of myeloid cells. These myeloid cells are immature and capable of helping the tumor to suppress the immune system, but can also promote the proliferation of new blood vessels, helping tumor growth. Therefore, the presence of a high number of macrophages in the sample is associated with a poor prognosis. The mechanisms behind these processes require further investigation [44].

The influx of macrophages changes irrespective of the treatment approach (radiotherapy, proton beam radiotherapy, or transpupillary thermotherapy). The number usually increases after treatment, showing that the most important roles of this specific leukocyte type are phagocytosis and tissue repair. While this remains under investigation, it is accepted that the role of macrophages and the presence of leukocytes in the uveal melanoma tumor microenvironment are important in determining patient outcomes. Furthermore, the presence of macrophages and other antigen-presenting cells in uveal melanoma is an important mediator of T-cell response and immunomodulation [44,45].

Another parameter that needs to be determined for cases of uveal melanoma is represented by the microvascularization and DD of the tumor. AJCC’s eighth edition states that microvascular density is associated with poor prognostic and metastatic risk. Vessels are quantified using immunostaining for CD31 and CD34, with their numbers determined in areas of highest vascularization (typical field corresponds to 0.31 mm^2^). DD represents the tumor’s basal diameter, which can be estimated based on the formula: 1 DD (optic disk diameter) = 1.5 mm. Thickness is estimated as 1 DD = 2.5 mm. Immunostaining for CD68 is recommended to assess macrophage density. Marker CD163 can also be used to determine cell quantity, categorized as few, moderate, or abundant. Higher numbers are associated with higher mortality. The mitotic count remains an important parameter for uveal melanoma. Mitoses are assessed by counting in 40 HPFs with a 40× objective, corresponding to a field area of 0.15–0.19 mm^2^ [28,44,46].

Given the dynamics between tumoral cells, immune cells, and treatment, immunotherapy can be considered a new step in managing patients with this pathology. One study described poor results when targeting PD-L1, suggesting that better options for cutaneous melanoma are needed [47]. Unfortunately, anti-BRAF therapy did not offer an alternative due to toxicity in both cutaneous and uveal melanoma [48,49].

All cases in this study were IHC stained for melanoma markers (SOX10, S100, Melan-A, and HMB45) and all were positive for primary cutaneous melanoma, uveal melanoma, and conjunctival melanoma. The malignant melanocytic cells in metastatic cases diffusely expressed *SOX10*. Despite this marker’s high specificity, several challenges must be considered. As previously highlighted by Scolyer, [50] *SOX10* can be expressed in regenerating Schwannian cells encountered post-excision. The most important differential diagnosis in such cases is desmoplastic melanoma, for which we must consider cytologic changes (nuclear atypia, nucleoli, and mitoses) and lesion architecture [51].

### Limitations

In light of our results, there are a few limitations that we should mention. These are represented by having a low number of cases for conjunctival melanoma and capsular nevus. The mentioned cases were the sole entities diagnosed in our praxis.

## 5. Conclusions

Melanoma remains a hot topic in cancer research since it is the most aggressive and frequent melanocytic malignant tumor. This study analyzed 100 melanoma cases and highlighted particularities, challenges, and additional pitfall diagnoses. The most common cases we encountered were nodular melanomas. Most cutaneous melanoma cases presented had stage pT4b. Prognostic factors (advanced stage, high Breslow index, and ulceration) were directly associated with metastases, leading to a poor prognosis. The most important tool in confirming the melanoma diagnosis was IHC with melanoma markers SOX10, S100, HMB45, and Melan-A. SOX10 shows high specificity, expressed in all primary and metastatic cases. Rare entities such as nevoid melanoma represent a diagnostic challenge requiring IHC confirmation. Capsular nevus represents a diagnostic pitfall that needs further investigation and guidelines. Uveal melanoma has new and unique independent prognostic factors that are assessed for diagnosis and outcome: macrophages, vascularization, and DD. Mitotic count for this tumor type is an independent factor related to metastatic risk, a parameter no longer used for cutaneous melanoma.

## Figures and Tables

**Table 1:** IHC markers.

Clone	Control and Expression	Stain Type
SOX10 rabbit monoclonal primary antibody, CELL MARQUE (SP267)	Control on melanocytes (basement layer), staining melanoma cells	Nuclear
S100 polyclonal primary antibody, VENTANA	Control on melanocytes (basement layer), staining melanoma cells	Nuclear
Anti-melanosome (HMB45) mouse monoclonal primary antibody, VENTANA	Control on melanocytes (basement layer), staining melanoma cells	Cytoplasmic
Melan-A A103, CONFIRM Anti-MART-1/Melan-A (A103) mouse monoclonal primary antibody, VENTANA	Control on melanocytes (basement layer), staining melanoma cells	Cytoplasmic
CONFIRM Anti-Ki-67 (30–9) rabbit monoclonal primary antibody, VENTANA	Keratinoblasts; expressed in >10% of melanoma cells	Nuclear

## Data Availability

Not applicable.
